# Supplementary material for: A methodology for the design of experiments in computational intelligence with multiple regression models
Source: PeerJ. 2016 Dec 1;4:e2721. doi: 10.7717/peerj.2721 (PMC5136129; doi:10.7717/peerj.2721)
Supplement: Data S1 — Datailed results from UC Irvine Machine Learning Repository (Housing, Machine CPU, Wine Quality, Automobile and Parkinson) and the 3 Use Cases (Protein Corona, Gajewicz Metal Oxides and Aquatic Toxicity) [file peerj-04-2721-s001.zip › 6_UseCases/1_ProteinCorona/LM.details.csv.repeatedcv.split1.pdf]

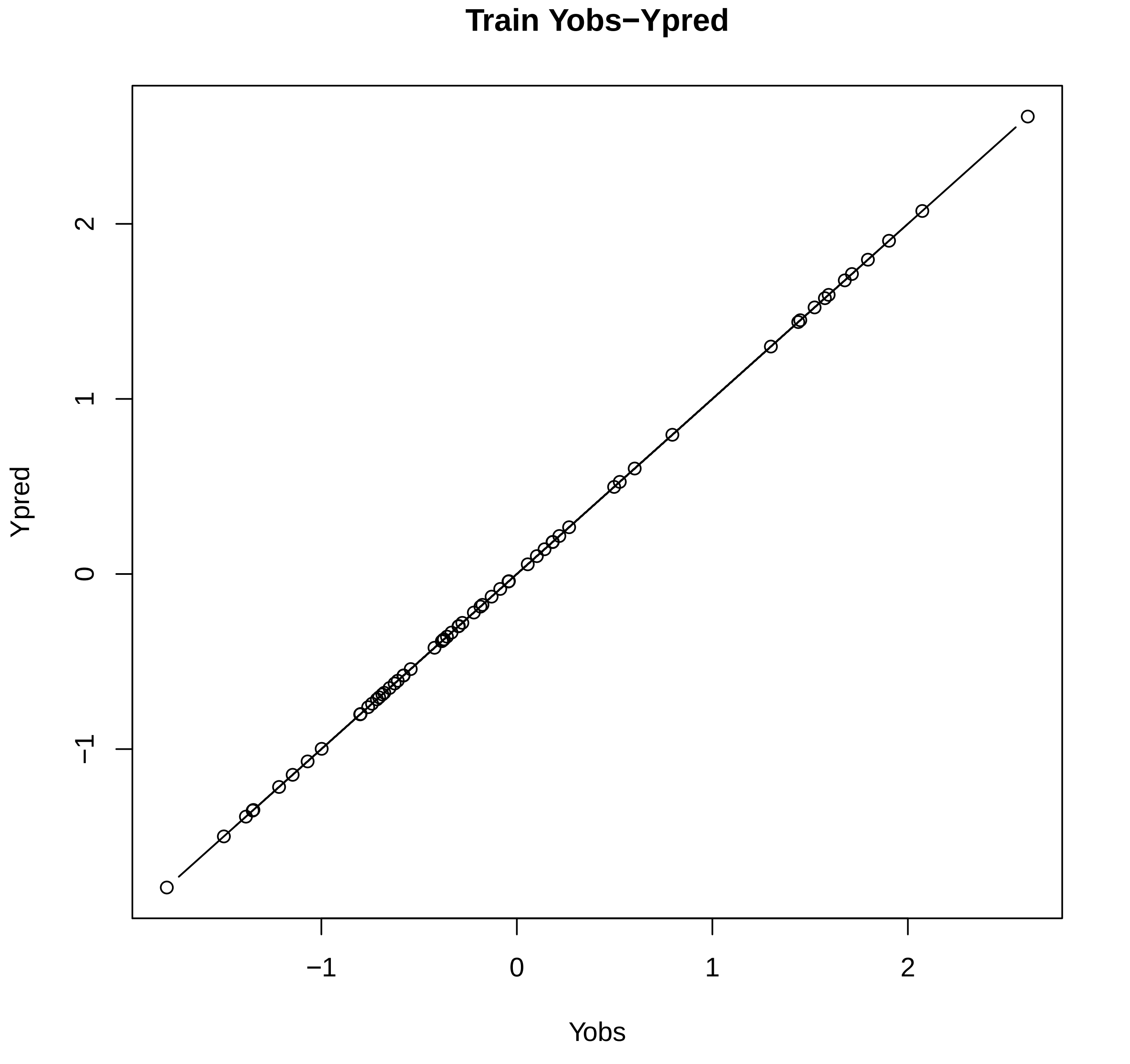

Train Yobs−Ypred
Ypred
Yobs
−1
0
1
2

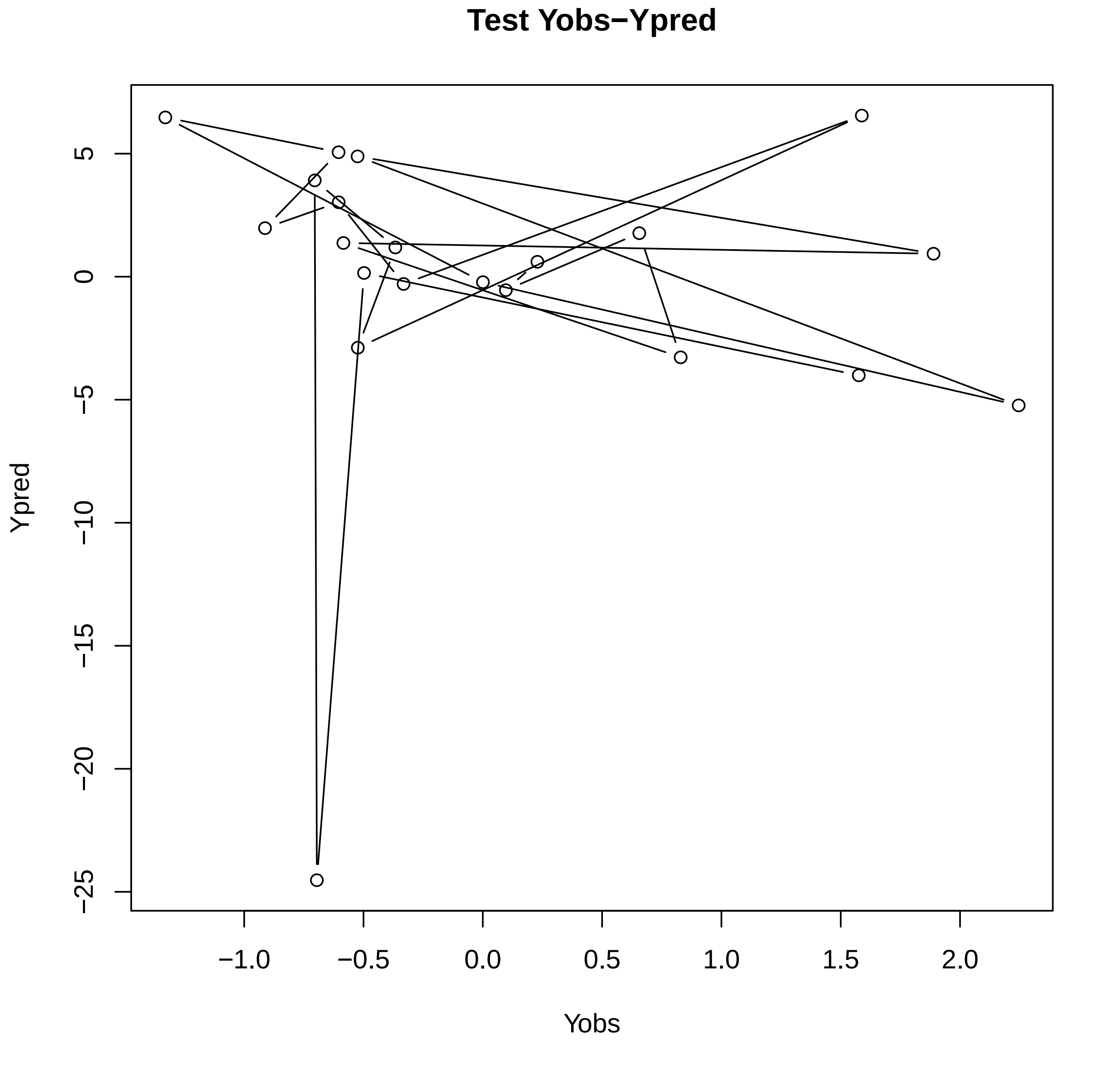
Test Yobs−Ypred
Ypred
Yobs
5
0
−5
−10
−15
−20
−25
−1.0
−0.5
0.0
0.5
1.0
1.5
2.0

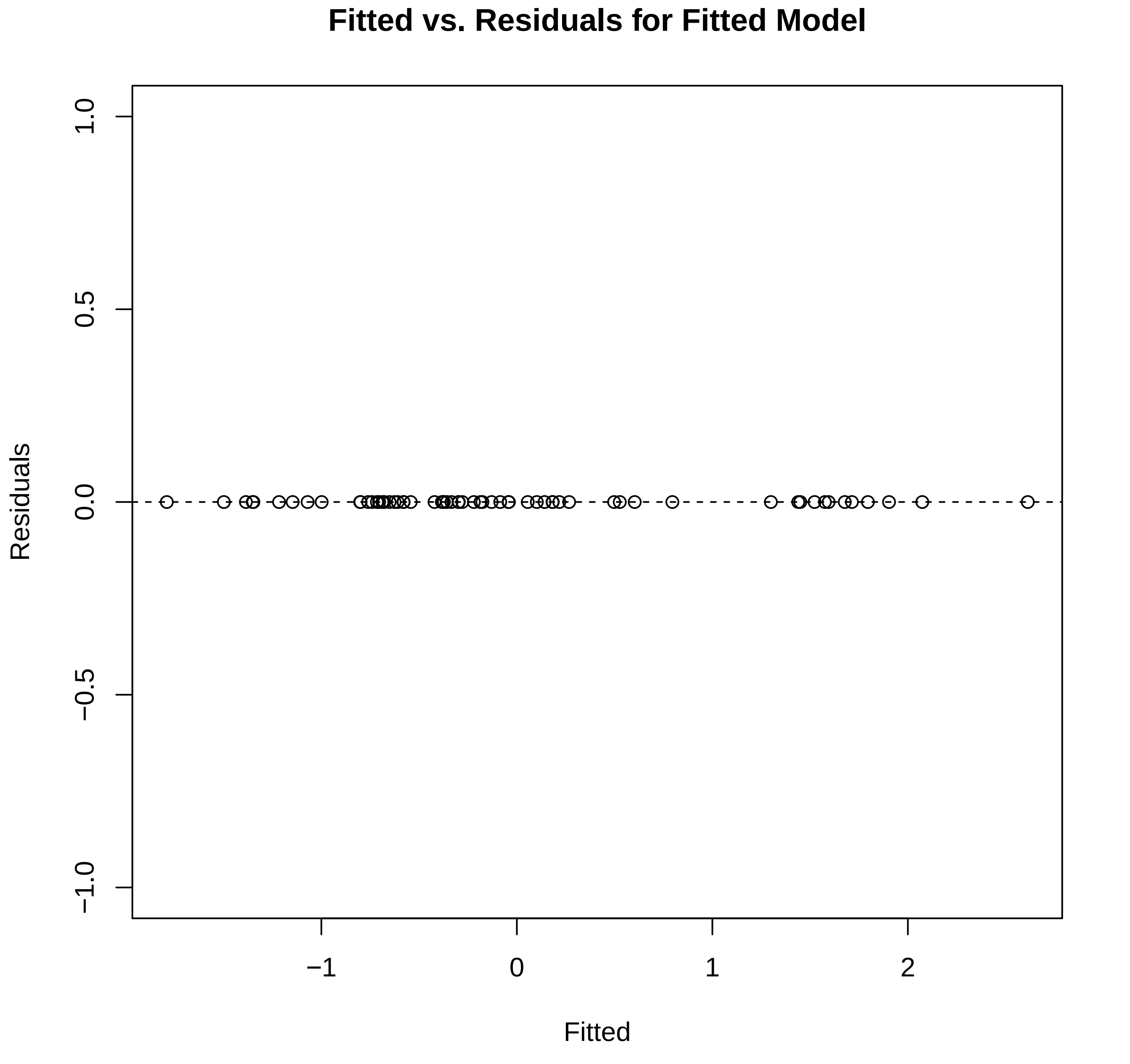
Fitted vs. Residuals for Fitted Model
Residuals
1.0
0.5
0.0
−0.5
−1.0
−1
0
1
2
Fitted

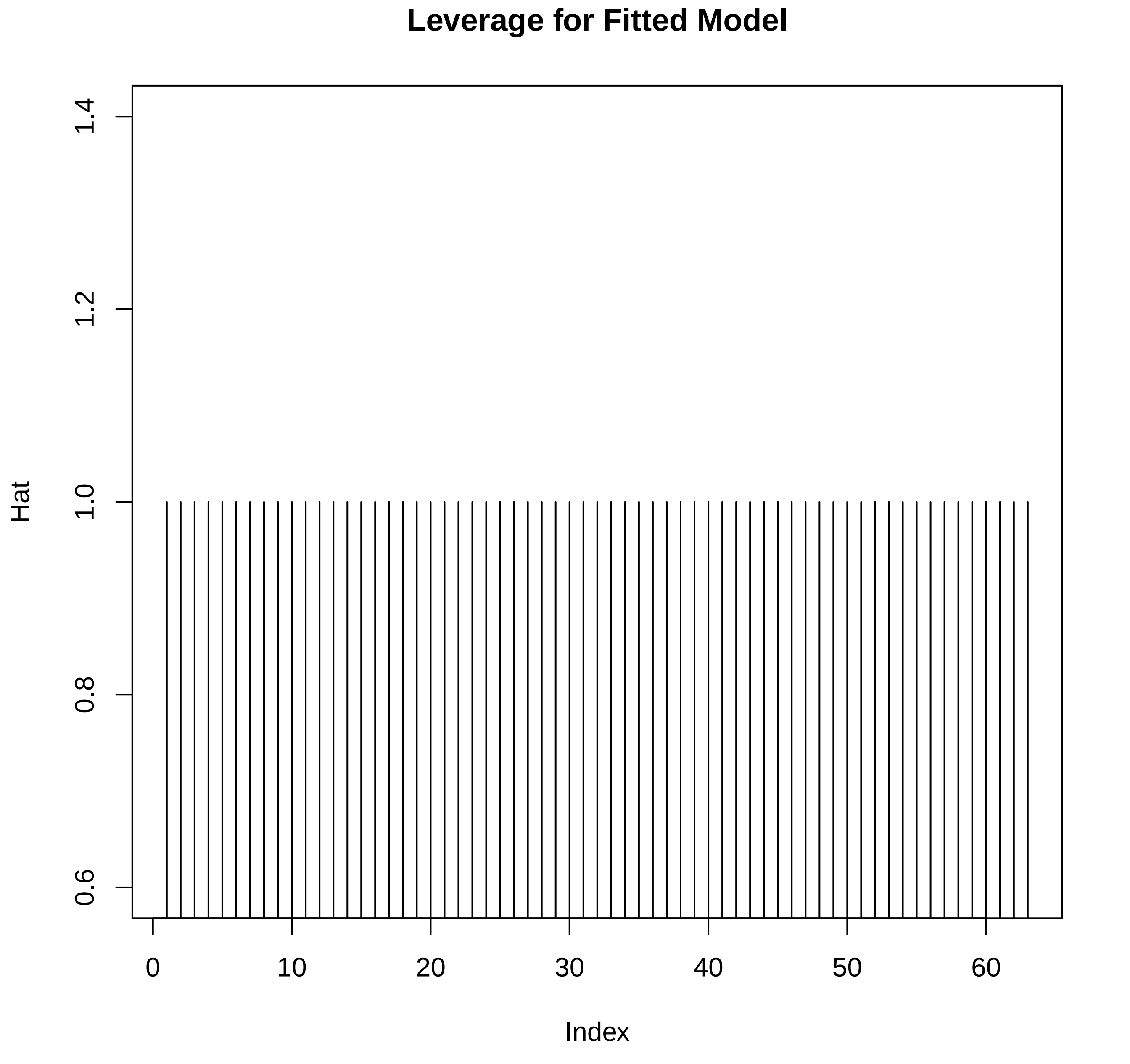
Leverage for Fitted Model
Hat
1.4
1.2
1.0
0.8
0.6
0
10
20
30
40
50
60
Index
